# Immediate and after effects of transcranial direct-current stimulation in the mouse primary somatosensory cortex

**DOI:** 10.1038/s41598-021-82364-4

**Published:** 2021-02-04

**Authors:** Carlos A. Sánchez-León, Isabel Cordones, Claudia Ammann, José M. Ausín, María A. Gómez-Climent, Alejandro Carretero-Guillén, Guillermo Sánchez-Garrido Campos, Agnès Gruart, José M. Delgado-García, Guy Cheron, Javier F. Medina, Javier Márquez-Ruiz

**Affiliations:** 1 Department of Physiology, Anatomy and Cell Biology, Pablo de Olavide University, Ctra. de Utrera, km. 1, 41013 Seville, Spain; 2 HM CINAC, Hospital Universitario HM Puerta del Sur, HM Hospitales, Madrid, Spain; 3 Instituto de Investigación E Innovación en Bioingeniería, Universidad Politécnica de Valencia, Valencia, Spain; 4 Laboratory of Electrophysiology, Université de Mons, Mons, Belgium; 5 Laboratory of Neurophysiology and Movement Biomechanics, ULB Neuroscience Institute, Université Libre de Bruxelles, Brussels, Belgium; 6 Department of Neuroscience, Baylor College of Medicine, Houston, TX USA

**Keywords:** Neurology, Neuroscience

## Abstract

Transcranial direct-current stimulation (tDCS) is a non-invasive brain stimulation technique consisting in the application of weak electric currents on the scalp. Although previous studies have demonstrated the clinical value of tDCS for modulating sensory, motor, and cognitive functions, there are still huge gaps in the knowledge of the underlying physiological mechanisms. To define the immediate impact as well as the after effects of tDCS on sensory processing, we first performed electrophysiological recordings in primary somatosensory cortex (S1) of alert mice during and after administration of S1-tDCS, and followed up with immunohistochemical analysis of the stimulated brain regions. During the application of cathodal and anodal transcranial currents we observed polarity-specific bidirectional changes in the N1 component of the sensory-evoked potentials (SEPs) and associated gamma oscillations. On the other hand, 20 min of cathodal stimulation produced significant after-effects including a decreased SEP amplitude for up to 30 min, a power reduction in the 20–80 Hz range and a decrease in gamma event related synchronization (ERS). In contrast, no significant changes in SEP amplitude or power analysis were observed after anodal stimulation except for a significant increase in gamma ERS after tDCS cessation. The polarity-specific differences of these after effects were corroborated by immunohistochemical analysis, which revealed an unbalance of GAD 65–67 immunoreactivity between the stimulated versus non-stimulated S1 region only after cathodal tDCS. These results highlight the differences between immediate and after effects of tDCS, as well as the asymmetric after effects induced by anodal and cathodal stimulation.

## Introduction

Transcranial direct-current stimulation (tDCS) is a safe and well tolerated neuromodulatory technique^1–4^ that relies on the application of constant weak electrical currents on the scalp during several minutes through strategically positioned electrodes^5,6^. Most studies using tDCS deliver a low-current intensity (from conventional 1–2 mA up to currents of 4 mA) between two rubber electrodes (25–35 cm^2^) placed on the scalp for 10–20 min^1,3,7^. Given its ability to modulate neuronal excitability, tDCS has attracted the attention of basic and clinical neuroscientists that have investigated its potential to modulate brain function^8^ and treat a variety of neurological conditions such as epilepsy^9^, attention deficit hyperactivity disorder (ADHD)^10^ or ataxia^11^ among others (for a review see^12–14^).

From a mechanistic point of view, the effects of tDCS on cortical excitability can be separated into immediate and after effects. Immediate effects, appearing at the very moment of electric field application, are related to changes in membrane polarization caused by redistribution of charges in the cells in presence of the externally applied electric field^15,16^. On the other hand, after effects observed following current cessation require several minutes of stimulation to develop and involve plasticity mechanisms^17^. Recently, *in vitro* models have been successfully used to show that different neuronal features such as the orientation of the somatodendritic axis with respect to the electric field^18^, the neuronal morphology^19^, or the axonal orientation^20^ are crucial to determine the overall immediate neuronal modulation, showing that purely depolarizing or purely hyperpolarizing stimulation does not exist^21^. In addition, animal and human studies have revealed that GABA levels^22–24^, glial cells^25^, neurotrophic BDNF^26^ and different receptors such as NMDA^27^, mGluR5^28^, AMPA^29,30^ and adenosine^31^ are involved in the long-term effects observed after tDCS. Thus, despite the simplicity of the technique, understanding the overall effect of transcranial electrical currents on brain tissue requires a comprehensive integration of several factors.

Previous tDCS studies have shown the ability of tDCS to modulate the amplitude and synchronicity of different EEG and LFPs frequencies in human subjects^32–34^ and more specifically, to modulate the amplitude of sensory-evoked potentials (SEPs) in both humans^35,36^ and animals^31^. SEPs are event-related potentials (ERPs) evoked by sensory stimulation^37^ and can be recorded in the human^38,39^ and rodent^40^ primary somatosensory cortex (S1), constituting a useful test bed for translational studies^41,42^.

The current study looks at polarity-specific immediate and after effects of tDCS applied to the mouse primary somatosensory cortex (S1-tDCS), addressing the electrophysiological and molecular changes caused by tDCS in the behaving mouse brain. To assess whether the effects of the stimulation were polarity-dependent^5^, we delivered either anodal or cathodal current. The neuromodulatory effects of S1-tDCS at the electrophysiological level were examined by recording the spontaneous LFPs and sensory-evoked potentials elicited by whisker electrical stimulation in S1 of alert mice. To identify molecular changes induced by S1-tDCS, we performed a post-stimulation immunohistochemical analysis of GAD 65–67 and vGLUT1 immunoreactivity in the stimulated brain region.

## Methods

### Animals

Experiments were carried out on adult males C57 mice (University of Seville, Spain) weighing 28–35 g. All experimental procedures were carried out in accordance with European Union guidelines (2010/63/CE) and following Spanish regulations (RD 53/2013) for the use of laboratory animals in chronic experiments. In addition, these experiments were submitted to and approved by the local Ethics Committee of the Pablo de Olavide University (Seville, Spain). This study was carried out in compliance with the ARRIVE guidelines.

### Surgery

**Figure 1:**
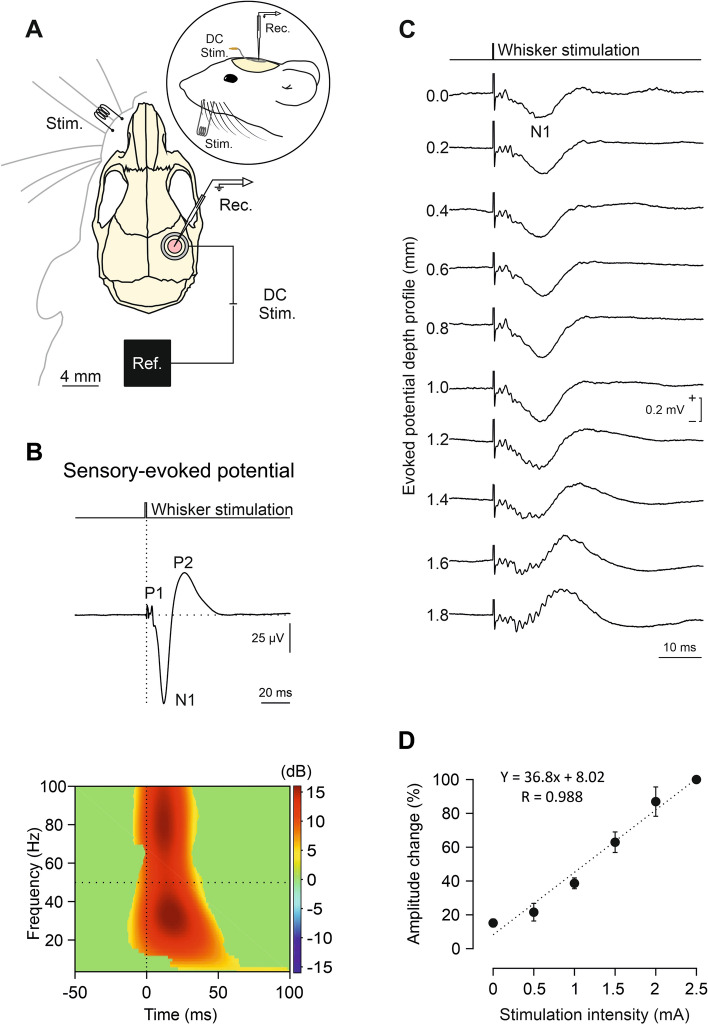
SEP characterization. (**A**) Experimental preparation for concurrent tDCS and *in vivo* electrophysiological recordings in S1. (**B**) (Up) Waveform showing the different components (P1, N1 and P2) and (down) ERSP of the SEP induced by whisker electrical stimulation (n = 10 mice). (**C**) S1-SEP depth profile. Every trace corresponds to an average of 10 SEPs recorded at different depths for a representative mouse. (**D**) Quantification of the amplitude change in N1 component of SEPs regarding intensities applied to whisker electrical stimulation. Data normalized with respect to maximum amplitude recorded at 2.5 mA (n = 3 mice).

Animals were prepared for chronic recording of SEPs and simultaneous transcraneal electrical stimulation (tES) following previously published work^43^. Animals were anesthetized with a ketamine–xylazine mixture (Ketaset, 100 mg/ml, Zoetis, NJ., USA; Rompun, 20 mg/ml, Bayer, Leverkusen, Germany) at an initial dosage of 0.1 ml/20 g. Under aseptic conditions, a custom-made silver ring chlorinated electrode (2.5 mm inner ø, 3.5 mm outer ø), which acted as the active electrode for tDCS, was placed over the skull centered on the right S1 vibrissa area (AP =  − 0.9 mm; Lateral = − 3 mm; relative to bregma^44^) (Fig. 1A) and covered with dental cement (DuraLay, Ill., USA). After that, a hole (2 mm ø) was drilled in the parietal bone inside the ring electrode to expose S1 and the dura mater surface was protected with wax bone (Ethicon, Johnson & Johnson, NJ., USA). In addition, a silver electrode was also implanted over the dura surface under the left parietal bone (AP =  − 0.9 mm; Lateral =  + 3 mm; relative to bregma^44^) as electrical reference for the electrophysiological recordings. To build the reference electrode, a silver wire (ø: 381 μm, A-M Systems) was cut into pieces of 1 cm length, then a loop (2 mm ø) was made at one end to facilitate posterior grasping by the amplifier equipment and the opposite end of the electrode was braided and filed to avoid damaging the dura mater. Regarding the histological experiments, the active electrode for tDCS consisted of a polyethylene tubing (outer ø: 3.25 mm; inner ø: 2.159 mm; A-M Systems) placed over the stimulated region and filled with electrogel in which the electrode from the stimulator was immersed. No trepanation was made in the histological experiments to avoid tissue damage. Finally, a head-holding system was implanted, consisting of three bolts screwed to the skull and a bolt placed over the skull upside down and perpendicular to the horizontal plane to allow for head fixation during the experiments. The complete holding system was cemented to the skull.

### Recording and stimulation procedures

Recording sessions began at least two days after surgery. The animals were placed over a treadmill with an infrared sensor for locomotion activity monitoring and the head was fixed to the recording table by means of the implanted head-holding system. To stimulate the whiskers, an electrical stimulus (0.2 ms square pulse, < 2.5 mA) was delivered through a pair of hook electrodes inserted in the left whisker pad connected to an isolation unit (CS20, Cibertec, Madrid, Spain) controlled by a stimulator device (CS420, Cibertec). To characterize the SEPs, a glass micropipette (1–5 MΩ of impedance; outer ø: 2.0 mm; inner ø: 1.6 mm; length: 15 cm, with inner filament; A-M Systems, WA., USA) was filled with 3 M NaCl, mounted on a micromanipulator (MO-10, Narishige, Tokyo, Japan) and placed over S1 area. In order to map the SEP, the electrical stimulus was delivered at the whisker pad every 10 ± 2 s, the micropipette was lowered and the current intensity adjusted (0.7–2.5 mA) until the maximum amplitude SEP was achieved. Then, the current intensity of whisker electrical pulses was lowered to elicit a SEP with half of the maximum amplitude to allow the observation of an increase or decrease of its components during and after tDCS intervention. All recordings were obtained with an amplifier (BVC-700A, Dagan corporation, MN., USA) connected to a dual extracellular-intracellular headstage (8024, Dagan corporation; gain error ± 1%, noise 4.5 µV root mean square) at a sampling rate of 25 kHz and an amplitude resolution of 12 bits (CED Micro 1401; Cambridge Electronic Design, Cambridge, UK).

### Transcranial electrical stimulation (tES)

The different protocols for transcranial currents were designed in Spike2 (Cambridge Electronic Design (CED), Cambridge, U.K.) and sent to a battery-driven linear stimulus isolator (WPI A395, Fl., USA). tDCS was applied between the ring electrode over S1 and a reference electrode consisting of a rubber rectangle (6 cm^2^) attached to the back of the mouse and moistened with electrogel (Electro-Cap International, OH., USA). A silver wire inserted into the rubber electrode allowed for stimulator connection. To measure the actual voltage changes elicited intracranially, transcranial alternating-current stimulation (tACS) was applied at three randomly interleaved intensities, ± 2, ± 20 and ± 200 μA (± 0.0426, ± 0.426 and ± 4.26 mA/cm^2^) at 1 Hz. To characterize the immediate effects induced by tDCS, 15 s pulses of anodal and cathodal tDCS were applied separated by 10 s of non-stimulation. To index after effects changes, tDCS was delivered during 20 min at 200 μA for cathodal stimulation, for 20 min at 150 μA for anodal stimulation and for 30 s at 150 μA anodal for sham stimulation. Differences in the intensity used for cathodal and anodal were due to amplifier noise issues with higher anodal currents. The current was directly monitored during experiments to ensure that it matched  the value indicated by the stimulator.

### Histology

To characterize potential histological changes associated to tDCS, a different set of animals received 20 min of anodal, cathodal or sham tDCS at 200 μA. 15 min after tDCS cessation mice were deeply anesthetized with ketamine–xylazine mixture (Ketaset, 100 mg/ml; Rompun, 20 mg/ml) and perfused transcardially with 0.9% saline followed by 4% paraformaldehyde (PanReac, Barcelona, Spain) in PBS. The brains were removed and stored in 4% paraformaldehyde for 24 h, cryoprotected in 30% sucrose in PB the next 48 h, and then cut into 50 μm coronal slices with a freezing microtome (CM1520, Leica, Wetzlar, Germany). After three washes of 10 min with PBS, sections were blocked with 10% Normal Donkey Serum (NDS, 566,460, Merck, Darmstadt, Germany) in PBS with 0.2% Triton X-100 (Sigma-Aldrich, Mo., USA) (PBS-Tx-10% NDS) and then incubated overnight at room temperature in darkness with mouse anti-vesicular Glutamate Transporter 1 (vGLUT1, 1:1000, MAB5502, Merck) or rabbit anti-Glutamate Decarboxylase 65–67 (GAD 65–67, 1:1000, AB1511, Merck). After three washes, sections were incubated for 1 h at room temperature in darkness with appropriate secondary antibodies: Alexa Fluor 488 donkey anti-mouse IgG (H + L) (1:400, A21202, Thermo Fisher Scientific, Mass., USA), Alexa Fluor 555 donkey anti-rabbit IgG (H + L) (1:400, A31572, Thermo Fisher Scientific) in PBS-Tx-5% NDS. After three washes with PBS, sections were mounted on glass slides and coverslipped and confocal images were acquired with a confocal microscope (A1R HD25, Nikon, Tokyo, Japan).

### Data analysis

To estimate the electric field strength during tACS, peak-to-peak value (electric potential) from the LFP evoked by tES was measured and averaged for a given intensity and depth. For each intensity the electric field strength (differences between potentials) was calculated by computing the difference in peak-to-peak values between two consecutive depths (1 mm in distance).

SEP amplitude was computed by the peak-to-peak command in Spike2 software, where the maximum negative voltage value (N1) was subtracted from the maximum positive voltage value (P1) of the preceding peak. SEPs recorded when the animal was running were removed from the analysis as well as those potentials presenting electrical artifacts.

Confocal images were processed in Fiji (http://fiji.sc/Fiji) using a custom built macro. Fluorescence background was subtracted and five square ROI of 100 × 100 pixels (291.31 μm^2^) were randomly placed over regions absent of nuclei or unspecific noise (as for example blood vessels). Each image inside the ROI was converted to binary and the “Analyze Particles” command was used to count and measure aggregates of vGLUT1 and GAD 65–67. Particles were averaged to obtain one value per hemisphere per animal.

ERP analysis was performed in EEGLAB rev.14.1.2 toolbox using Matlab 2015a software package. Data were segmented, baseline was corrected by subtracting the mean voltage level in the first 500 ms interval of the window and artifacts produced by electrical stimulation in the whiskers eliminated^45^. Data were averaged for each condition and subject, to obtain the SEP by using the electrical stimulation as a trigger in EEGLAB toolbox and temporal periods were statistically compared.

To analyze the spectral dynamics of the neural oscillations (Fast Fourier Transform—FFT) and Event-Related spectral perturbation (ERSP), an analysis of the *induced activity* was performed. For that, average SEP from every subject was subtracted from each condition, temporal period and subject^46,47^.

FFT from every subject was extracted from each condition and temporal period and averaged. FFTs from 20 min post-tDCS and 40 min post-tDCS were compared with the FFT from 20 min of control condition for every condition independently. A statistical analysis by permutations (*p* < 0.05) with a false discovery rate (FDR) for multiple comparisons was applied.

A time–frequency signal analysis was performed trial-by-trial using Hanning-windowed sinusoidal wavelets at 1 cycle (lowest) to 13.3 cycles (highest). Changes in event-related dynamics of the signal spectral power were studied using the ERSP index^48^. Significance thresholds for ERSP were calculated by a bootstrap distribution (*p* < 0.05), extracted randomly from the baseline data (from − 330 to 0 ms) and applied 400 times^49^. Additionally, the ERSP of the different temporal periods were statistically compared by permutations analysis (*p* < 0.05).

SigmaPlot 11.0 (Systat Software Inc, San Jose, CA., USA), IBM SPSS version 25 (IBM, Armonk, NY)) and Matlab 2015a (MathWorks Inc.) were used for statistical analysis. Normality was assessed using the Shapiro–Wilk test (*p* value > 0.05). For immediate effect experiments, statistical significance of differences between groups was inferred by a two-way repeated-measures analysis of variance (ANOVA), with CURRENT INTENSITY (50, 100, 150 or 200 μA) and POLARITY (anodal or cathodal) as within-subject factors, and the post hoc Holm-Sidak test for multiple comparisons. For after effects experiments, a two-way repeated-measures ANOVA was performed to infer statistical differences with TIME (temporal periods of 5 min each: one time point for control, four time points during tDCS/sham and twelve time points after tDCS/sham) as within-subject factor, and tDCS POLARITY (anodal, cathodal or sham) as between-subjects factor. The post hoc Bonferroni test was applied for multiple comparisons. For immunohistochemical experiments, statistical comparison for fluorescence levels was inferred by a two-way mixed ANOVA with BRAIN HEMISPHERE (non-stimulated vs. stimulated hemisphere) as within-subject factor and tDCS POLARITY (anodal, cathodal or sham) as between-subjects factor. The post hoc Bonferroni test was applied for multiple comparisons. The results are shown as mean ± SEM. Statistical significance was set at *p* < 0.05 in all cases.

## Results

### Characterization of sensory-evoked potentials in response to whisker stimulation

To index potential changes in the neuronal excitability of S1 during and after tDCS, SEPs in response to whisker stimulation were chronically recorded in alert head-restrained mice (n = 10; Fig. 1A). Electrical whisker stimulation evoked a contralateral short-latency SEP in the vibrissa S1 area (Fig. 1B) consisting of a first positive component (P1) peaking at 3.8 ± 0.2 ms (n = 10), followed by a negative wave (N1) at 12.6 ± 1.2 ms (n = 10), and finally a positive slower component (P2) peaking at 26.2 ± 2.8 ms (n = 10). The amplitude and latency of the N1 component of SEP varied along the recording sites across cortical layers (Fig. 1C), reaching maximum amplitude between 0.8 – 1.0 mm depth and showing a polarity inversion at deeper recording sites. The final amplitude of the N1 component was linearly dependent on the intensity of the electrical stimuli applied to the whiskers, as shown in Fig. 1D (R = 0.988; *p* < 0.001; n = 3). Finally, the ERSP of SEPs (Fig. 1B, at the bottom) was characterized by a significant increase in power spectrum for all analyzed frequencies (3–100 Hz) associated with the first 50 ms of SEP after the whisker stimulation. As observed in Fig. 1B, two major frequency bandwidths were maximally enhanced, one at 20–40 Hz and other at 60–100 Hz.

### tACS-elicited electric field decays with distance from the active electrode

**Figure 2:**
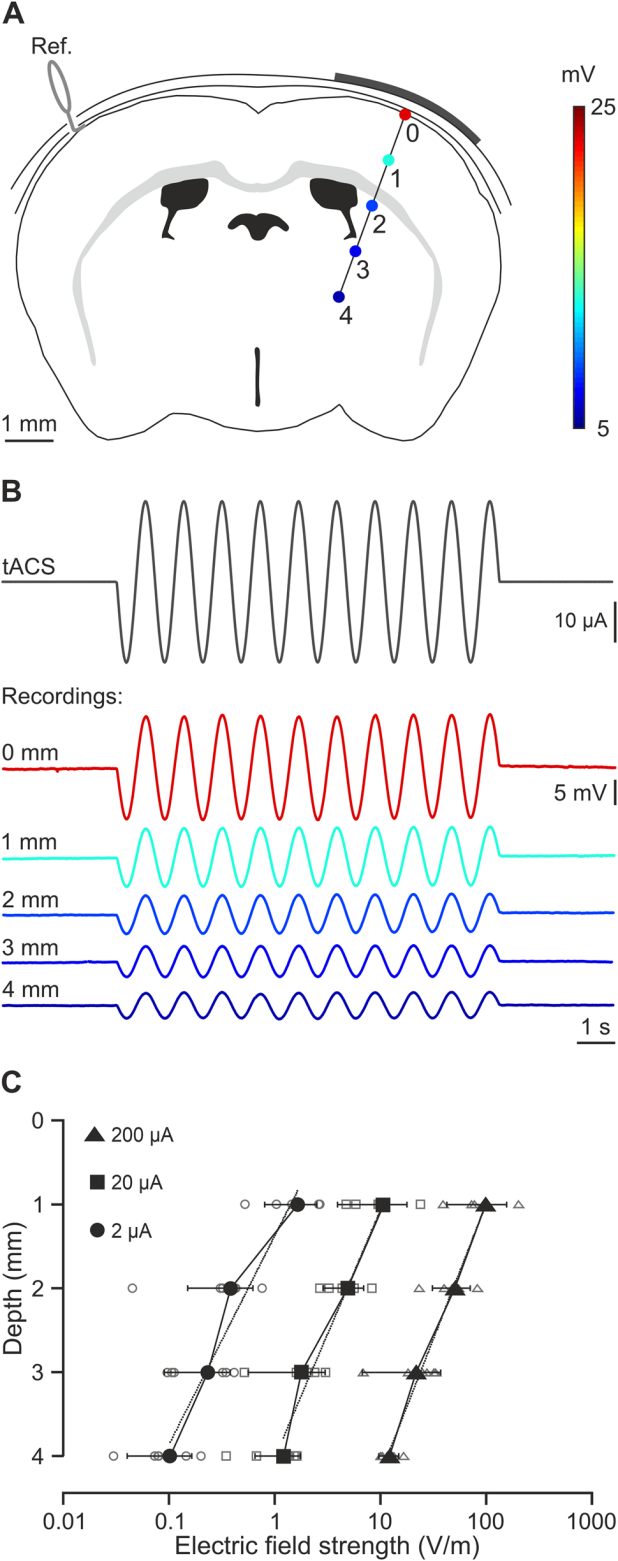
Intracranial electric fields induced by S1-tDCS. (**A**) Schematic representation of electric potentials recorded in S1 at different depths. (**B**) tACS stimulation (top trace) applied over the scalp and grand average (n = 6 mice, unprocessed data) of the actual potentials generated at different depths (from 0 to 4 mm). (**C**) Average (filled symbols) and individual (empty symbols) electric fields recorded at different depths for ± 2 (circles), ± 20 (squares) and ± 200 μA (triangles) tACS.

In a first experiment, we determined the actual electric field gradient along the brain tissue imposed by transcranial electrical stimulation (tES) application in our experimental design. Animals (n = 6) were prepared for chronic recording of LFPs in the S1 area in alert condition during simultaneous application of low-frequency tACS (1 Hz) (Fig. 2A). Differential recordings were sequentially performed every 1 mm from the cortical surface to 4 mm depth. Figure 2B shows the grand average obtained from recordings at different depths including the data from all six animals. The calculated electric fields at different depths and intensities are represented in Fig. 2C. Under the active electrode, the magnitude of the electric field decreased with depth in a logarithmic manner for the three tested intensities (data are presented with logarithmic abscissa axis for visual facilitation, Fig. 2C). To calculate the electric field imposed by tES at different intensities in the recording site (~ 1 mm) we used a linear regression equation extracted from the relation between tACS intensity and electric field strength (E = − 0.4473 * I − 0.731; R = 0.997; *p *= 0.0028; n = 3).

### tDCS increases and decreases the amplitude of simultaneously recorded SEPs in a polarity and intensity-dependent manner

**Figure 3:**
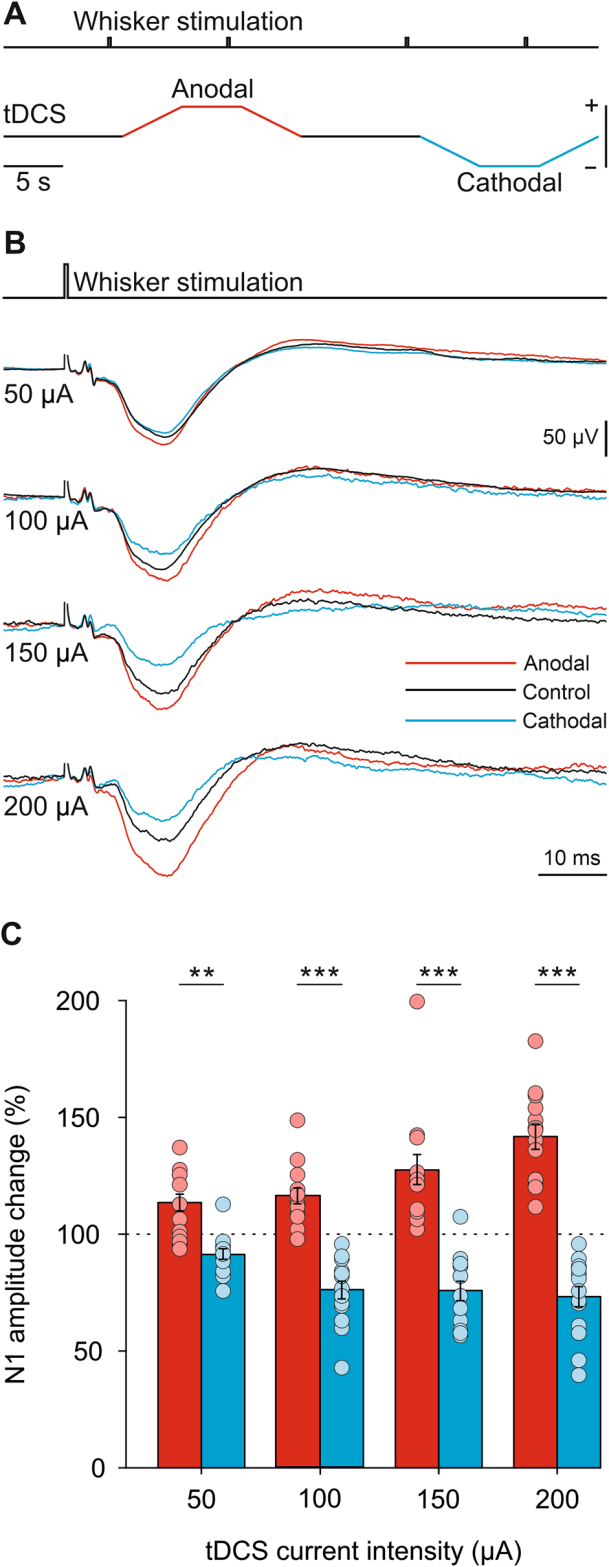
tDCS immediate effects over SEPs in S1 cortex. (**A**) Schematic representation of tDCS protocol. (**B**) SEP average (n = 30) from a representative animal during control (black trace), anodal (red trace) and cathodal (blue trace) tDCS applied at 50, 100, 150 and 200 μA. (**C**) Quantification and statistical results of anodal tDCS (red histograms) and cathodal tDCS (blue histograms) effects on SEP amplitude. Mean (bars) and individual amplitude data (circles) are represented as percentage of change with respect to control values for all animals (n = 14 mice). Two-way repeated-measures ANOVA, CURRENT INTENSITY effect, F_3,39_ = 3.316, p = 0.030, POLARITY effect, F_1,13_ = 51.081, *p* < 0.001, CURRENT INTENSITY × POLARITY interaction, F_3,39_ = 27.818, *p* < 0.001, Holm-Sidak post hoc test. **p < 0.01; ***p < 0.001.

To test immediate effects of tDCS on S1 excitability we recorded SEPs induced by whisker pad stimulation during simultaneous short-duration [15 s, including 5 s ramp up and 5 s ramp down (Fig. 3A)] anodal and cathodal tDCS pulses, at 4 randomly interleaved intensities (50, 100, 150 and 200 µA). The calculated electric field strength induced by 50, 100, 150 and 200 μA at the recording site was 23.1, 45.5, 67.8 and 90.2 V/m, respectively. SEPs recorded just before tDCS pulses were used as controls, to normalize the peak-to-peak amplitude in anodal and cathodal conditions. Figure 3B shows the averaged SEPs (n = 30) during the control condition (black trace), anodal (red trace) and cathodal (blue trace) tDCS for a representative animal. Mean data obtained from the group of animals participating in the experiment (n = 14) are represented in Fig. 3C. Thus, increasing the intensity of anodal tDCS progressively increased (to a maximum of 141.5 ± 5.3% at 200 µA) the amplitude of simultaneously recorded SEPs with significant differences for all intensities compared to cathodal situation, whereas increasing the intensity of cathodal tDCS significantly decreased the SEPs amplitude to a minimum of 73.2 ± 4.4% at 200 µA (two-way repeated-measures ANOVA, CURRENT INTENSITY: F_3,39_ = 3.316, *p* = 0.030; POLARITY: F_1,13_ = 51.081, *p* < 0.001, interaction CURRENT INTENSITY × POLARITY: F_3,39_ = 27.818, *p* < 0.001; Holm-Sidak, *p* < 0.05; Fig. 3C). In summary, the magnitude and direction of tDCS effects on the N1 amplitude of simultaneously recorded SEPs were dependent on the applied polarity and intensity.

### tDCS induces asymmetric after effects on SEP amplitude depending on different current polarity

**Figure 4:**
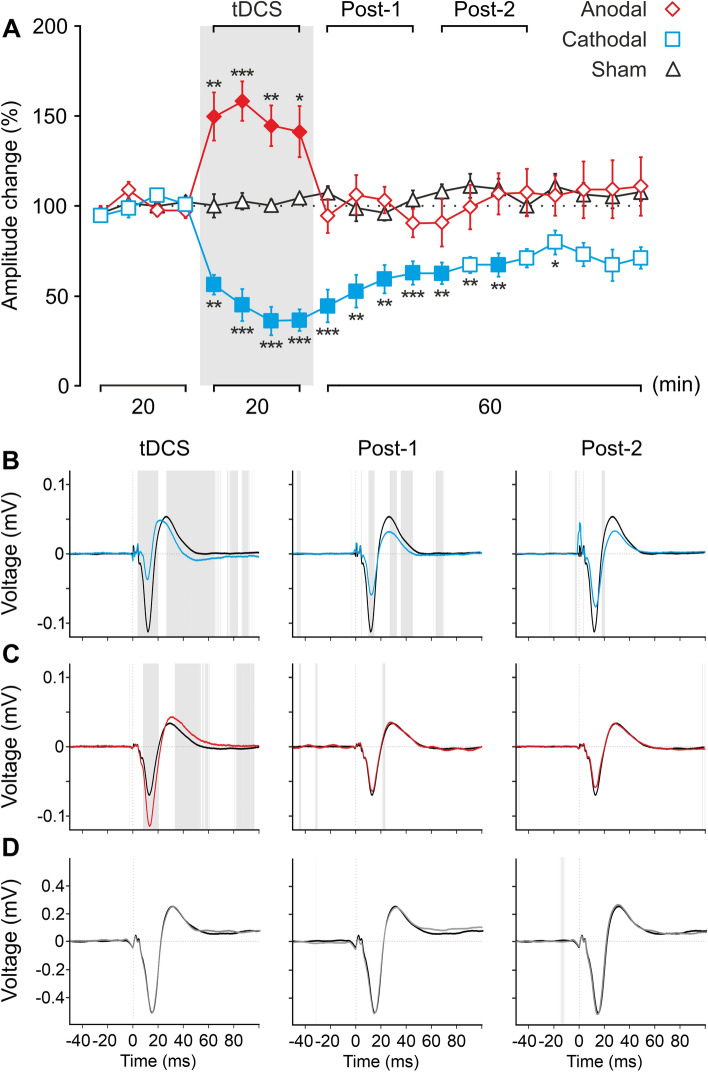
tDCS after effects over SEPs in S1 cortex. (**A**) Normalized amplitude change of N1 averaged every 5 min for 20 min of anodal (red diamonds), cathodal (blue squares) or sham (black triangles) tDCS. Two-way repeated-measures ANOVA, TIME: F_5.4,146.5_ = 2.792, p = 0.016; tDCS POLARITY: F_2,27_ = 20.895, *p* < 0.001, interaction TIME × tDCS POLARITY: F_10.9,146.5_ = 8.967, *p* < 0.001. Filled symbols represent statistical differences with the last control period (n = 10 mice, *p* < 0.05, Bonferroni post hoc test). Asterisks mark statistical differences between the same temporal period for anodal or cathodal with sham tDCS (n = 10 animals, *p < 0.05; **p < 0.01; ***p < 0.001, Bonferroni). (**B-D**) ERP analysis comparing all SEPs averaged 20 min before tDCS (black trace) with respect to averaged SEPs during 20 min of tDCS (first column), or averaged SEPs in the first 20 min after tDCS cessation (Post-1, second column) or the next 20 min (Post-2, third column) for cathodal (blue traces), anodal (red traces) or sham (grey traces) tDCS. Grey shadow represents statistical differences between points (*p* < 0.05, paired t-test).

To test potential after effects of tDCS over S1 excitability we recorded SEPs induced by whisker pad stimulation (every 10 ± 2 s) in three different randomly assigned experimental conditions, anodal (n = 10), cathodal (n = 10) or sham (n = 10) group. During experimental sessions SEPs were recorded for 20 min before tDCS, during continuous anodal (150 µA, 20 min), cathodal (200 µA, 20 min) or sham (150 µA, 30 s) tDCS, and for 1 h after tDCS. Every 5-min interval SEP waveforms were averaged and normalized with respect to baseline values (control condition, before tDCS). As observed in Fig. 4A, tDCS has a significant effect on the normalized N1 amplitude of SEPs for both anodal and cathodal polarity (two-way repeated-measures ANOVA, TIME: F_5.4,146.5_ = 2.792, *p* = 0.016; POLARITY: F_2,27_ = 20.895, *p* < 0.001, interaction TIME × POLARITY: F_10.9,146.5_ = 8.967, *p* < 0.001; Bonferroni, *p* < 0.05). Interestingly, anodal tDCS significantly increased the amplitude of SEPs (up to a maximum of 158.2 ± 11.0%, n = 10) with respect to control values only during simultaneous tDCS intervention (Bonferroni, *p* < 0.05; red filled diamonds in Fig. 4A), whereas cathodal tDCS decreased the amplitude of SEPs with respect to control values reaching its maximum effects (maximum of 33.1 ± 7.9%, n = 10) during simultaneous tDCS application remaining significantly decreased for 25 min after tDCS cessation (Bonferroni, *p* < 0.05; blue filled squares in Fig. 4A). No significant effects were observed in the amplitude of N1 component of SEPs in the sham group (Bonferroni, *p* < 0.05; black triangles in Fig. 4A). As expected, significant differences between anodal and sham group were restricted to the tDCS period (Bonferroni, *p* < 0.05, Fig. 4A, asterisks) whereas significant differences were maintained during and for 35 min after tDCS when comparing the cathodal with the sham group (Bonferroni, *p* < 0.05, Fig. 4A, asterisks).

We also analyzed the grand average SEP waveforms induced by whisker pad stimulation (ERP analysis). As shown in Fig. 4B (left blue trace), cathodal tDCS significantly decreased the amplitude of different components in the simultaneously recorded SEPs (gray shading indicates *p* < 0.05, n = 10, Fig. 4B). This effect was progressively reduced after cathodal tDCS cessation, being maintained during the first 20 min (middle Fig. 4B) and almost inexistent for the next 20 min period (right Fig. 4B). On the other hand, anodal tDCS significantly increased the amplitude of different components in the simultaneously recorded SEPs (gray shading indicates *p* < 0.05, n = 10, Fig. 4C) whereas no remarkable significant effects were observed 20 min (middle Fig. 4C) or 40 min (right Fig. 4C) after anodal tDCS. As expected, no remarkable significant effects were observed in the sham group (n = 10, Fig. 4D).

**Figure 5:**
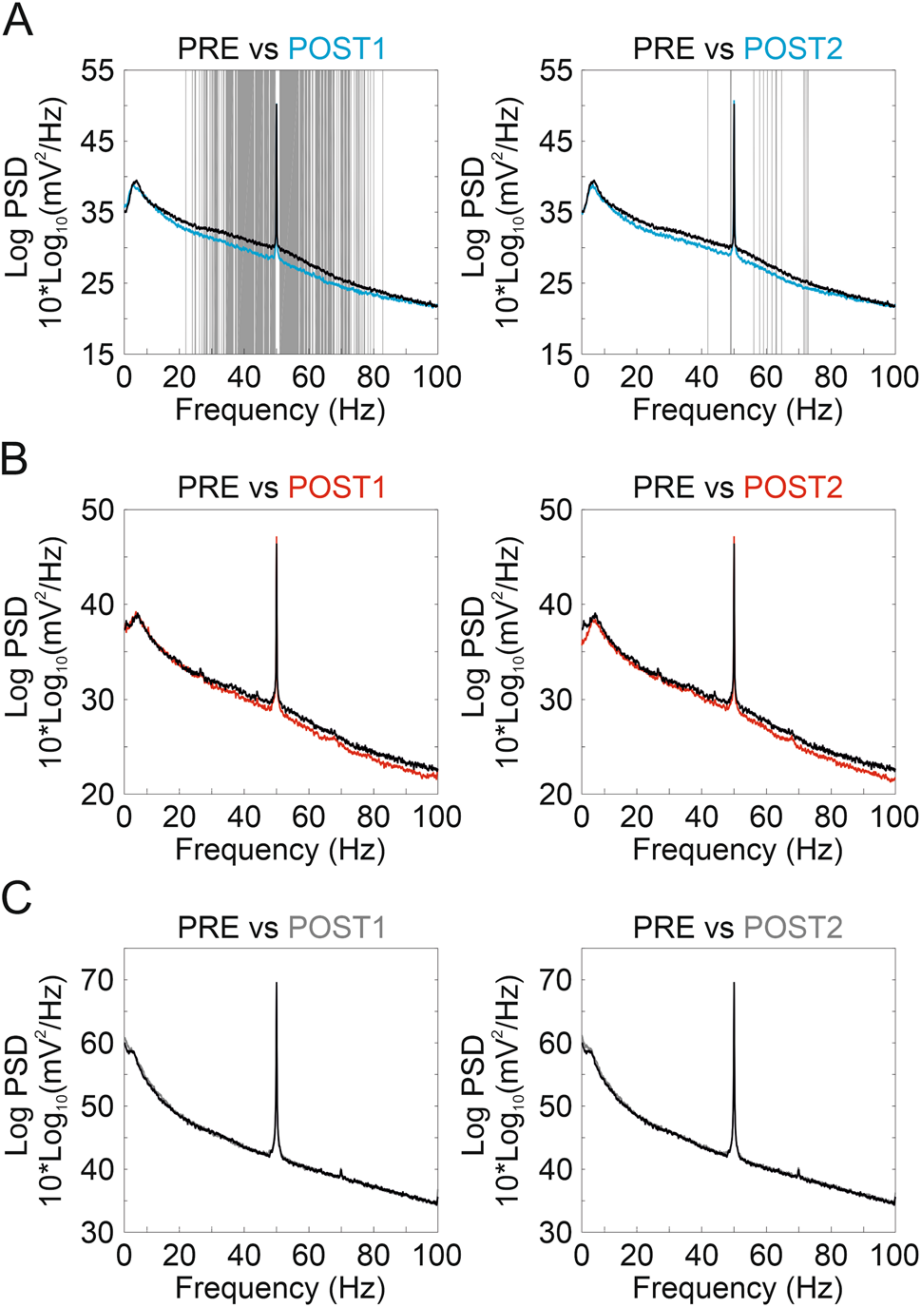
FFT analysis. (**A–C**) Comparison between the FFT obtained 20 min before the stimulation and the first 20 min after tDCS cessation (POST1, first column) or the next 20 min (POST2, second column) for cathodal (**A**), anodal (**B**) or sham (**C**) tDCS. Grey shadow represents statistical differences for a specific frequency (*p* < 0.05, permutations).

We analyzed the potential significant changes in the power spectrum of the induced activity (selecting a temporal window of ± 2.5 s with respect to the whisker stimulus) before (PRE) and after (first 20 min: POST1; and next 20 min: POST2) cathodal (Fig. 5A), anodal (Fig. 5B) tDCS and sham condition (Fig. 5C). No CONTROL vs. DURING temporal periods were analyzed in this case because tDCS-associated artifacts were often present in the selected time intervals (5 s duration). Significant differences (permutation analysis with FDR for multiple comparisons, *p* < 0.05, n = 10 for anodal and sham, n = 9 for cathodal condition) were observed between POST1 and PRE condition in the 20–80 Hz band for cathodal tDCS (Fig. 5A, left column, gray shading indicates *p* < 0.05) showing a decrease in the amplitude of cortical oscillations in this bandwidth. These differences were not present (except for a few points in the 50–70 Hz range) when PRE and POST2 were compared (Fig. 5A, right column, gray shading indicates *p* < 0.05). Interestingly, there were no significant changes in any of the comparisons after anodal tDCS (Fig. 5B) or sham condition (Fig. 5C).

**Figure 6:**
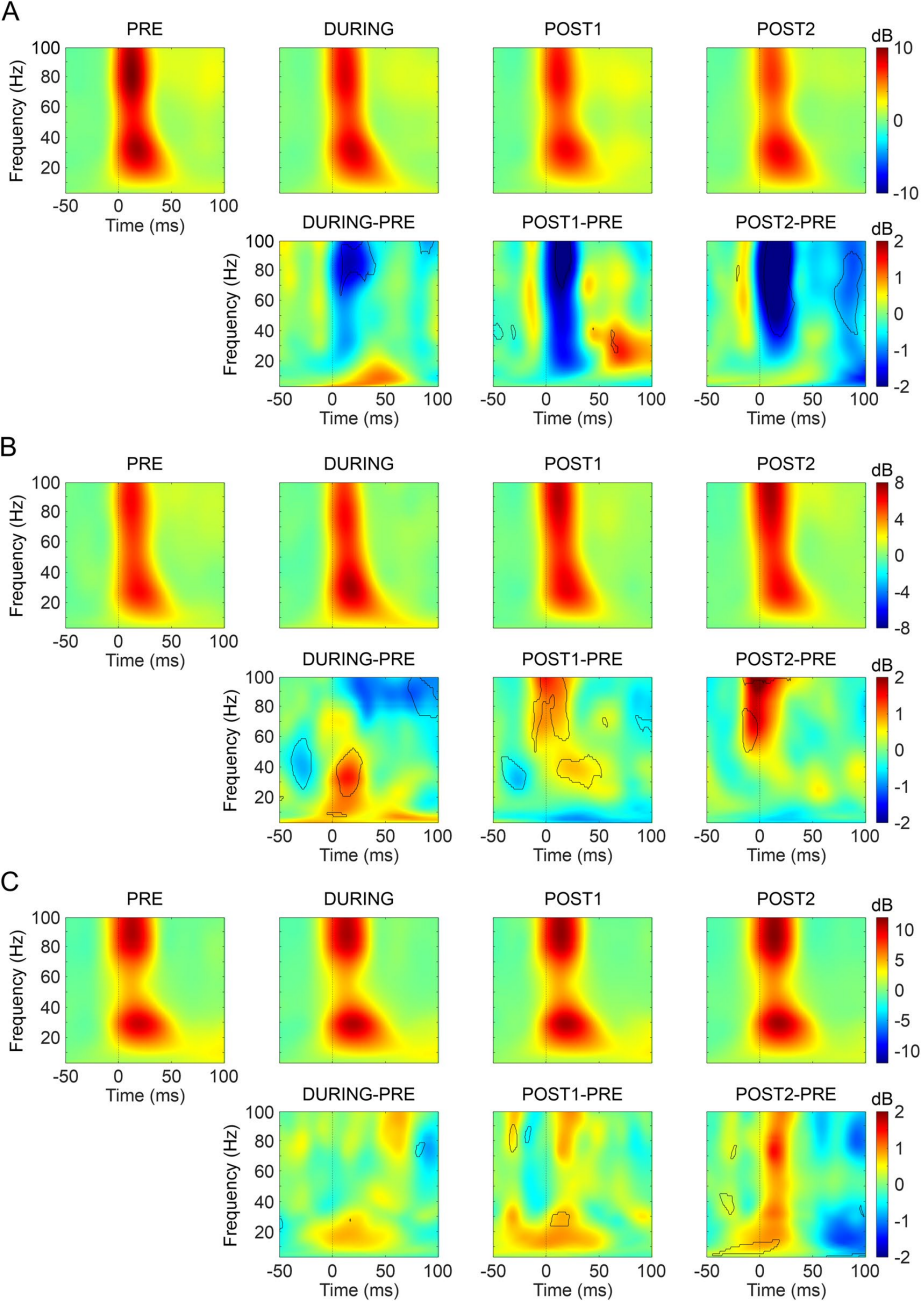
ERSP analysis of the induced activity. (**A–C**) (Upper row) ERSP obtained for the 20 min before stimulation (PRE, first column), during tDCS (DURING, second column), the first 20 min after tDCS cessation (POST1, third column) and the next 20 min (POST2, fourth column) for cathodal (**A**), anodal (**B**) or sham (**C**) tDCS. (Lower row) Differences between the ERSP obtained in the 20 min before stimulation (PRE) and DURING (first column), POST1 (second column) or POST2 (third column) for cathodal (**A**), anodal (**B**) or sham (**C**) tDCS. Black outline represents statistical differences for that frequency and time range (*p* < 0.05, permutations).

To know more about the organization of these frequency differences in the temporal domain we carried out a spectral dynamic analysis of induced response (from 50 ms before to 100 ms after whisker stimulation) associated to sensory stimulation before, during and after tDCS (Fig. 6). During the 20 min of cathodal tDCS we found a significant decrease in the spectral power covering the bandwidth between 70 and 100 Hz (0–40 ms), also during 20 min after tDCS (POST1-PRE in Fig. 6A) and extended to lower frequencies (40–100 Hz; 0–40 ms and 70–100 ms) for the last 20 min (POST2-PRE in Fig. 6A) (permutation analysis, *p* < 0.05, n = 10; indicated by cooler colors in DA maps DURING-PRE). In contrast, during 20 min of anodal tDCS intervention the spectral power was significantly higher (permutation analysis, *p* < 0.05, n = 10, indicated by warm colors in DA maps) with respect to the control period (DURING-PRE in Fig. 6B) covering a bandwidth between 20 and 50 Hz (0–20 ms). Unlike the results of the SEP analysis, where no differences were obtained after anodal tDCS (Fig. 4A,C), we found an increase in the spectral power corresponding to the time frame after whisker stimulation (> 0 ms) at two bandwidths between 30 and 50 Hz (10–50 ms) and 60–100 Hz (0–20 ms) throughout the 20 min after anodal tDCS (POST1-PRE in Fig. 6B) with only a few significant changes in the following 20 min (POST2-PRE in Fig. 6B). With respect to the time frame previous to the whisker stimulation (< 0 ms) the spectral power decreased in the 30–60 Hz bandwidth (− 40 to − 20 ms) during anodal tDCS intervention and throughout the 20 min after stimulation, and increased in the 50–80 Hz bandwidth (− 20 to 0 ms) in the whole period following tDCS removal (POST1-PRE and POST2-PRE in Fig. 6B). There were no significant changes in the sham group (n = 10) except for small-scattered differences (Fig. 6C).

### Cathodal tDCS induces GAD 65–67 but not vGLUT1 changes in S1 cortex

**Figure 7:**
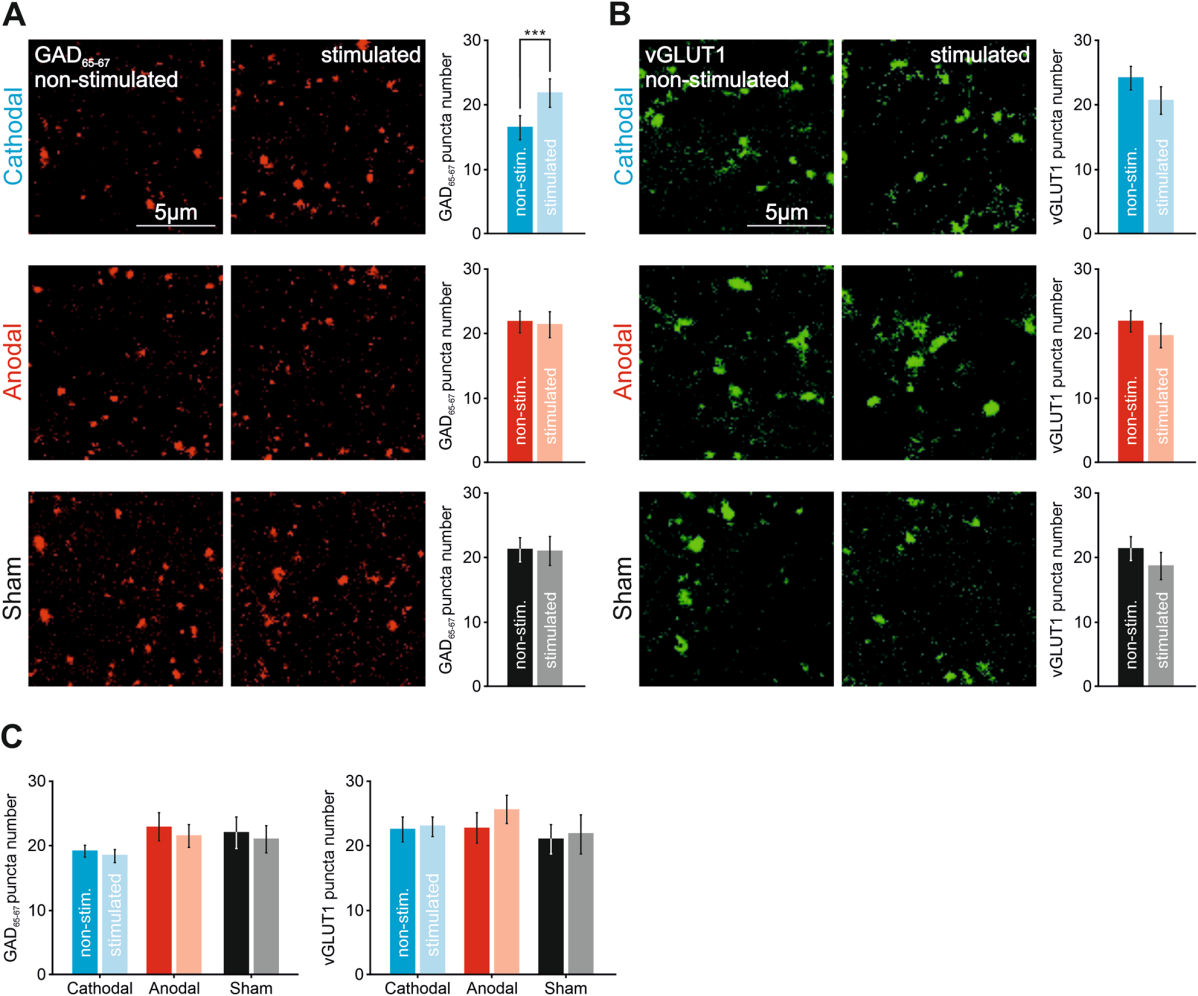
Immunohistochemical changes after 20 min of S1-tDCS. (**A,B**) Confocal photomicrographs (images), quantification and statistics (bar charts) of GAD 65–67 (**A**) or vGLUT1 immunoreactivity (**A**) in S1 after 20 min of cathodal tDCS (upper row, n = 4 mice, two-way mixed ANOVA, F_2,10_ = 5.163, ***p < 0.001), after 20 min of anodal tDCS (middle row, n = 5 mice) and after 20 min of sham condition (lower row, n = 4 mice). (**C**) Same analysis but for adjacent motor cortex. Error bars represent SEM. GAD 65–67: Glutamic acid decarboxylase isoforms 65 and 67; vGLUT1: vesicular glutamate transporter 1; OD: optical density; IR: immunoreactivity; A.U.: arbitrary units.

To elucidate potential molecular expression changes underlying the observed asymmetric after effects of anodal versus cathodal tDCS, we used antibodies against vGLUT1 and GAD 65–67 to assess possible modifications of the excitation/inhibition balance in the transcranially stimulated S1. A group of animals prepared for tDCS application during whisker stimulation (no electrophysiological recordings were carried out in this experiment) was randomly assigned to anodal (n = 5), cathodal (n = 4) or sham (n = 4) condition. Representative confocal images from the non-stimulated left hemisphere and the transcranially stimulated right hemisphere are presented in Fig. 7 for cathodal (at top), anodal (at middle) and sham (at bottom) groups for GAD 65–67 (Fig. 7A) and vGLUT1 (Fig. 7B). The number of GAD 65–67 and vGLUT1 positive clusters of puncta in the stimulated and non-stimulated S1 were analyzed in the cathodal, anodal and sham groups. We obtained a significant general main effect on the GAD 65–67 positive clusters for the interaction BRAIN HEMISPHERE × tDCS POLARITY (two-way mixed ANOVA, F_2,10_ = 5.163, *p* = 0.029, Fig. 7A) with a significant difference between the stimulated vs. non-stimulated hemisphere in the cathodal tDCS condition (Bonferroni, *p* = 0.005, Fig. 7A) indicating a higher number of GAD 65–67 positive clusters in the stimulated S1 hemisphere than in the non-stimulated S1. There was no significant difference in vGLUT1 between the stimulated and non-stimulated hemisphere in any of the tested stimulation conditions (two-way mixed ANOVA, F_2,10_ = 0.12, *p* = 0.888, Fig. 7B). No significant differences were found for anodal or sham condition. Finally, to exclude possible spreading effects of cathodal tDCS in the mouse cortex we also tested GAD 65–67 and vGLUT1 positive clusters in the adjacent primary motor cortex (M1). No significant effects were found in any GAD 65–67 and vGLUT1 positive clusters (two-way mixed ANOVA, F_2,10_ = 0.06, *p* = 0.942, Fig. 7C) suggesting a focalized histological after effect of cathodal tDCS on the stimulated region.

## Discussion

The present electrophysiological and histological results point to asymmetric differences between immediate and after effects observed during and after anodal or cathodal S1-tDCS. These results could be of crucial importance for human tDCS protocols suggesting that tDCS polarities could differently impact on cortical excitation/inhibition balance during or after its application.

The first aim of this study was to directly quantify the strength of the induced electric field at different cortical depths. As expected, we observed bigger electric field values in the first millimeter of the cortex following a logarithmic decay with increasing distance. In line with previous studies performed in humans and non-human primates electric fields generated during tES behave in a linear ohmic manner^50^. However, in the present study the electric field (23.1–90.2 V/m) imposed on the cortical layer of the mice, resulted to be considerably higher than those typically used in humans (1 V/m)^50,51^. Interestingly, previous investigations showed that electric field intensities used in humans generally fail to generate neuronal modulation when applied to animal models *in vivo*^52,53^. A possible explanation for this divergence could be related to differences in axonal lengths^54^ or larger neuronal densities found in primates^55^. In addition, the size of the electrodes used in human experiments, covering large cortical regions, could be optimizing neuronal recruitment by increasing network emergent effects^56,57^.

Our second aim was to investigate immediate effects of tDCS over the S1 mouse cortex. Our results indicate that tDCS applied for several seconds over S1 is sufficient to modulate cortical excitability in agreement with previous results reported from the human motor cortex^5^. We observed an increase of SEP amplitude during anodal stimulation and a decrease during cathodal stimulation. Furthermore, tDCS modulated cortical excitability in an intensity-dependent manner, i.e. greater intensities induced greater changes. Previously, our team has reported similar results in S1 cortex from rabbits in response to air-puff whisker stimulation or ventroposterior medial (VPM) thalamic nucleus stimulation during simultaneous tDCS and slow-frequency (0.05 Hz) tACS^31,58^. Comparable results have also been observed in motor^59^ and visual^25,60^ cortices in mice. Overall, these results point toward a similar immediate effect of tDCS across different cortices, at least for the simplified cortical geometry of mice and rabbits where the axo-dendritic orientation of pyramidal cells with respect to the exogenous electric field is homogeneous^18–20^.

Our third aim was to explore whether tDCS induces after effects in SEP amplitude. Unlike immediate effects, a long-lasting modulation of SEP amplitude was only observed after cathodal tDCS, with no changes after anodal tDCS. Similar asymmetric results have been previously reported by our team in alert rabbits where cathodal but not anodal S1-tDCS was able to induce after effects measured by a reduction of SEP amplitude after the stimulation^31^. The present study extends our previous observations in the rabbit tDCS model^31^, by investigating the impact of tES on cortical oscillatory activity and by assessing histological changes related to glutamate and GABA neurotransmission. Specifically, this new study shows novel data about tDCS effects not only after 20 min of transcranial stimulation but during tDCS including also a new sham group which received only 30 s tDCS (Fig. 4). In addition, we performed new electrophysiological experiments aimed to characterize actual current diffusion across cortical layers (Fig. 2) (as far as we know there are no previous studies that have reported something similar in awake rodents). In the current study we show new original results describing the impact of tDCS on the SEP waveform (ERP in Fig. 4), the power spectrum of the induced activity (FFT in Fig. 5) and spectral dynamic analysis of induced response (ERSP in Fig. 6). These last analyses (ERP, FFT and ERSP) are all new with respect to our previous observations regarding the impact of tDCS in the rabbit model and because they are commonly used in human basic and clinical electrophysiology we think they are valuable from a translational point of view. Finally, immunohistochemistry results on glutamate and GABA markers support the electrophysiological observations (Fig. 7) constituting a novel approach that has not been performed in our previous study in rabbits^31^. Human studies have also reported diverging effects of tDCS. First of all, a few studies have reported a similar absence of effects after anodal tDCS intervention in humans^36,61^. Whereas in other studies performed in humans anodal S1-tDCS increased the amplitude of somatosensory evoked magnetic fields^38^ and improved performance in a complex somatosensory task after current application^62^. In conformity with our results, reported effects after stimulation in humans indicate that cathodal S1-tDCS besides decreasing tactile perception^61^, reduced SEP amplitude^36,39^ in correlation with increasing sensory and pain thresholds^39^. The reported similarities between mice, rabbits and humans by using the same electrophysiological biomarker (SEP) point to a common mechanism for tDCS on S1. The asymmetry of after effects observed in the present study is an important issue since long-lasting excitability changes are crucial for clinical treatments^13^. Moreover, the lack of after effects after anodal stimulation suggests that anodal tDCS may be most effective when applied online, during a given task, rather than before or after it^13,63^.

An additional approach of the present study to further explore long-lasting effects of tDCS has been the power spectrum analysis. In this regard, analysis of the power spectrum not in phase with the sensory events (FFT) showed that only cathodal (but not anodal) tDCS affected the amplitude of oscillations (ranging from 20 to 80 Hz) throughout the 20 min after transcranial stimulation. Moreover, sensory event-related spectral dynamics analysis showed that cathodal tDCS was able to decrease the spectral power in a wide range of frequencies (60–100 Hz) during the intervention as well as for up to 20 min after the stimulation, and in the range between 50 and 100 Hz for up to 40 min after tDCS. On the other hand, anodal tDCS increased the range between 20 and 50 Hz during stimulation, and 30–50 Hz and 60–100 Hz for up to 20 min after the stimulation. Thus, the application of tDCS on S1 seems to modulate gamma activity both during and after transcranial intervention. Accordingly, tDCS intervention in humans has shown to modulate brain oscillations at different frequencies and cortical regions. Specifically, cathodal tDCS caused a significant decrease of spontaneous and induced gamma in the occipital cortex^32,34^. On the other hand, anodal tDCS increased spontaneous theta and alpha frequency powers in prefrontal and occipital cortices^34,64^, and induced beta and gamma in occipital cortices posterior to the stimulation^32^. Interestingly, gamma oscillations have been related to visual attention^65,66^, codification, retention and retrieval of information independently of sensory modality^46,67,68^ together with sensory perception^69^. Overall, our finding suggests that tDCS may provide an effective method to modulate a variety of cognitive functions^70^.

Finally, to examine potential after effects in glutamate and GABA expression associated to anodal and cathodal tDCS we used antibodies against vGLUT1 and GAD 65–67. tDCS induced a GABA level imbalance between the stimulated and non-stimulated hemisphere after cathodal stimulation but no changes for anodal stimulation. This result is in line with our electrophysiological measures, suggesting an overall decrease in the excitability of the stimulated cortex after cathodal tDCS, but no long-lasting effects after anodal tDCS. In humans, polarity-dependent effects on GABA and glutamatergic levels after M1-tDCS have been reported^22^, indicating a relation between long-lasting tDCS effects and the cortical excitation/inhibition balance^71^. Some studies have shown a decrease in GABA after anodal M1-tDCS in the stimulated site^22–24^ and in the non-stimulated M1^23^. Interestingly enough, Bachtiar and colleagues (2018) describe a decrease in GABA only in the non-stimulated M1 after cathodal stimulation^23^, similar to what we observed after cathodal tDCS in our experiments. Further molecular and cellular analysis, together with a detailed electrophysiological examination of changes in excitatory and inhibitory synaptic transmission, will be necessary to corroborate the explorative results shown in the present study.

According to our results, optimal selection of tDCS parameters should be based on extensive knowledge of the brain mechanisms underlying the immediate and after effects of exogenous electric fields at single cell, synaptic, and network levels^21,72,73^. These different mechanisms could explain the heterogeneous effects of tDCS reported in human subjects that administered various protocol parameters implicating the position of stimulating electrodes over the scalp, the polarity, duration and density of the current^74^. Nevertheless, the complication of recording the actual electric field generated inside the human brain, together with the common use of indirect measurements of cortical excitability^7^, makes it difficult to examine tDCS-associated mechanisms in human studies. On the other hand, tES applications in animal models present important differences with respect to tES interventions in humans. Anatomical aspects such as the lack of cortical circumvolutions (in rodents) or a smaller brain size and thinner cortical thickness should be taken into account before basic research findings can be translated to the clinic^42,75^. Examining the impact of tES on the activity of neural networks^76,77^ and electrical field distribution^50^ in the brains of non-human primates could help bridge the difference between animal and human work.

In summary, the present electrophysiological and immunohistological study clearly shows differences between immediate and after effects of tDCS on S1, besides a distinct functional asymmetry in anodal and cathodal associated to the after effects. The complexity of the reported effects highlights the importance of defining both the immediate and after effects of tDCS on neural processing, to help improve stimulation protocols for treating neurological disease in the clinic.
